# Fathers’ Views and Experiences of Creating a Smoke-Free Home: A Scoping Review

**DOI:** 10.3390/ijerph16245164

**Published:** 2019-12-17

**Authors:** Rachel O’Donnell, Kathryn Angus, Peter McCulloch, Amanda Amos, Lorraine Greaves, Sean Semple

**Affiliations:** 1 Institute for Social Marketing, Faculty of Health Sciences and Sport, University of Stirling, Stirling FK9 4LA, Scotland, UK; 2 GRIT, Usher Institute, University of Edinburgh, Edinburgh EH8 9AG, Scotland, UK; 3 Centre of Excellence for Women’s Health, Vancouver, Canada & School of Population and Public Health, University of British Columbia, Vancouver, BC V6R 1Z3, Canada

**Keywords:** scoping review, barriers, facilitators, fathers, males, gender, smoking, smoke-free home, second-hand smoke

## Abstract

Enabling parents to create a smoke-free home is one of the key ways that children’s exposure to second-hand smoke (SHS) can be reduced. Smoke-free home interventions have largely targeted mothers who smoke, and there is little understanding of the barriers and facilitators that fathers experience in creating a smoke-free home. Systematic searches combining terms for fathers, homes, and SHS exposure were run in April 2019 in Web of Science’s Citation Indices, PsycINFO, and PubMed for English-language studies published since 2008. The searches identified 980 records for screening, plus 66 records from other sources. Twelve studies reported in 13 papers were included in this scoping review. Eight of the studies were conducted in Asian countries (five in China, one in India, one in Japan, and one in Iran), three were conducted in Canada, and one in Turkey. Findings were extracted in verbatim text for thematic analysis. The review identified that attitudes and knowledge, cultural and social norms, gender power relations, and shifting perceptions and responsibilities related to fatherhood can impact on fathers’ views of their role in relation to creating and maintaining a smoke-free home. There were too few published studies that had assessed smoke-free home interventions with fathers to draw conclusions regarding effective approaches. Research is clearly needed to inform our understanding of fathers’ roles, successes and challenges in creating and maintaining a smoke-free home, so that father-inclusive rather than mother-led interventions can be developed to benefit entire households and improve gender equity as well as health.

## 1. Introduction

Governments, health practitioners, and wider society all have a duty to protect non-smokers from the harms caused by second-hand smoke (SHS) exposure, which is estimated to cause nearly 900,000 deaths per annum and approximately 0.7% of global morbidity [1]. With substantial progress made in introducing smoke-free legislation in many countries in the past decade, most children’s exposure to SHS now occurs in their own home [2], with 40% of children worldwide regularly exposed to SHS indoors [3]. Studies conducted in Japan [4], the USA [5], Australia [6], Germany [7], and Denmark [8] have documented social disparities in children’s exposure to SHS at home, with children living in socio-economic disadvantage more likely to be exposed than children living in more affluent areas. In Scotland, 15% of children living in the most deprived areas are still exposed to SHS in their homes, compared to only 1% in the most affluent areas [9].

In the UK and elsewhere, smoke-free homes research has largely focused on the role of women and mothers in creating a smoke-free home [10], and the barriers and facilitators associated with women’s (mother’s) experiences of smoking behaviour change in these settings. There is a lack of available data estimating the proportion of fathers globally who smoke in the home, and little is known about their roles in creating and maintaining a smoke-free home, despite evidence that, with few exceptions, men are more likely to smoke than women [11]. These differences are particularly stark in East Asia and the Pacific region, where current figures suggest 49% of men smoke compared to less than 3% of women [12]. In households where relationships are vulnerable, gender power imbalances are strongly evident, with studies citing women’s lack of agency in effecting change in male smoking behaviours in their relationships or household [10]. On this basis, there have been recent calls in China and Malaysia for smoke-free home interventions to be delivered at a household level, rather than specifically targeting mothers [13,14], highlighting the need for approaches that engage with all members of smoking households.

Developing smoke-free home interventions that work directly with fathers, rather than tasking mothers with reminding, persuading, or negotiating with fathers to take their smoking outside the home, could address gender-specific issues underlying fathers’ smoking in the home as well as relieving mothers of this burden. It would also frame household smoking as a household responsibility, with family-wide impact. The call to include gender in tobacco control dates back 40 years to the 1980s–90s [15,16]. Gender-sensitive approaches have recently been used in Canada to develop father-friendly smoking cessation interventions [17,18] that are sensitive to gender-related factors that may influence the approach and outcomes [19]. Gender-transformative approaches go one stage further, applying gender theory in designing tobacco cessation/reduction initiatives with the dual aim of changing negative gender and social norms, and improving health and gender equity [20]. This goal explicitly aims to shift societal-level gender norms and stereotypes for both men and women in order to improve health, and in particular, to achieve equitable health opportunities for both men and women [21].

The aim of this scoping review was to synthesize findings on (1) the barriers and facilitators associated with changing fathers’ smoking behaviour in the home, and (2) the development, delivery, and effectiveness of interventions aimed at changing fathers’ smoking behaviour in the home.

## 2. Materials and Methods

A scoping review was carried out as they are increasingly used to examine the extent, range, and nature of existing research on a given topic or question. Scoping reviews are also used to identify gaps in the literature, and aid in the planning of future research [22,23], which also guided our choice of review method. In reporting this review, we have been guided by the PRISMA extension for scoping reviews (PRISMA-ScR) checklist [23]. A review protocol is available from the authors on request. 

Studies were eligible for the scoping review if they met the following inclusion criteria:

- Populations: the study’s sample comprised fathers, step-fathers, or male partners who smoked and lived in a home where a wife/partner and/or children also either lived or spent time there as their home, herein referred to as fathers.
- Interventions and Comparisons: the study could include none or any intervention and none or any comparison.
- Outcomes: the study investigated smoking behaviour in the home (evidenced by self-report and/or changes in objective measures of exposure to SHS (air quality, biological markers); and/or changes in SHS attitudes and/or knowledge; and/or any barriers and facilitators to changing smoking behaviour or creating a smoke-free home.
- Study types: the study collected qualitative and/or quantitative primary data, was written in English, and published since January 2008. This time frame was selected to limit the search to contemporary studies, and to acknowledge potential shifts in attitudes to smoking and smoke-free home environments associated with the increased focus on introducing comprehensive smoke-free laws from 2005 since the entry into force of the WHO Framework Convention on Tobacco Control (WHO-FCTC) [24].
- Studies of expectant fathers or female partners were also excluded because pregnancy is a well-documented ‘teachable moment’ where women may be more motivated to stop smoking [25]. Although a smaller number of studies have examined the extent to which pregnancy is a motivator for expectant fathers who quit smoking, it has been suggested that fathers are willing to make changes to their smoking behaviour during this time [26,27,28] and that they may feel differently about their health habits during pregnancy because they are more focused on the family as a whole [29].
- Grey literature and other literature reviews were also excluded.

A systematic search for studies was run in the following databases on 2 April 2019: Web of Science Citation Indices (Science Citation Index Expanded, Social Sciences Citation Index and Arts and Humanities Citation Index), PsycINFO, and PubMed. The search strategy combined terms for fathers, smoking/second-hand smoke, and homes, and was limited to English language records published since 1 January 2008 (see Appendix A for a sample search strategy). Other sources of papers were recommendations from academic experts; the reference lists of two relevant literature review papers [19,30]; and a search of Web of Science Citation Indices for smoking-related papers authored by Dr. J.L. Bottorff or Prof. J.L. Oliffe (see also Appendix A). Search results were downloaded to reference management software and duplicates excluded. Records were single-screened for inclusion on titles initially [KA], then potentially-relevant records were double-screened for inclusion by abstract [PM, RO]. Finally, full-text papers were double-screened for inclusion [PM, RO]. This double-screening process incorporated checks on our interpretation of, and agreement on, the barriers and facilitators we identified in existing studies. Any disagreements for inclusion were resolved by a third reviewer [KA], with the final set of included studies checked by members of the wider review team (See Supplementary Materials for a list of papers excluded at the full text screening stage).

Data were extracted by a single reviewer [PM] initially into a simple table to collect each study’s objective, sample, setting, country, study design, analysis method, intervention (if any), and the relevant findings. The latter were extracted in verbatim text from the papers’ results and discussion sections for analysis. All extractions were checked for accuracy by a second reviewer [RO]. No quality assessments were made of individual studies included in this review, as scoping reviews do not aim to produce critically appraised and synthesized results, and are used to provide an overview or map of the evidence in a given topic area [31]. Study findings were read and re-read by two reviewers initially to (a) identify broad themes that were then categorized as barriers or facilitators to fathers creating a smoke-free home, and (b) identify efforts to test smoke-free home interventions with a sample or sub-sample of fathers in discussion with the wider review team.

## 3. Results

**Figure 1:**
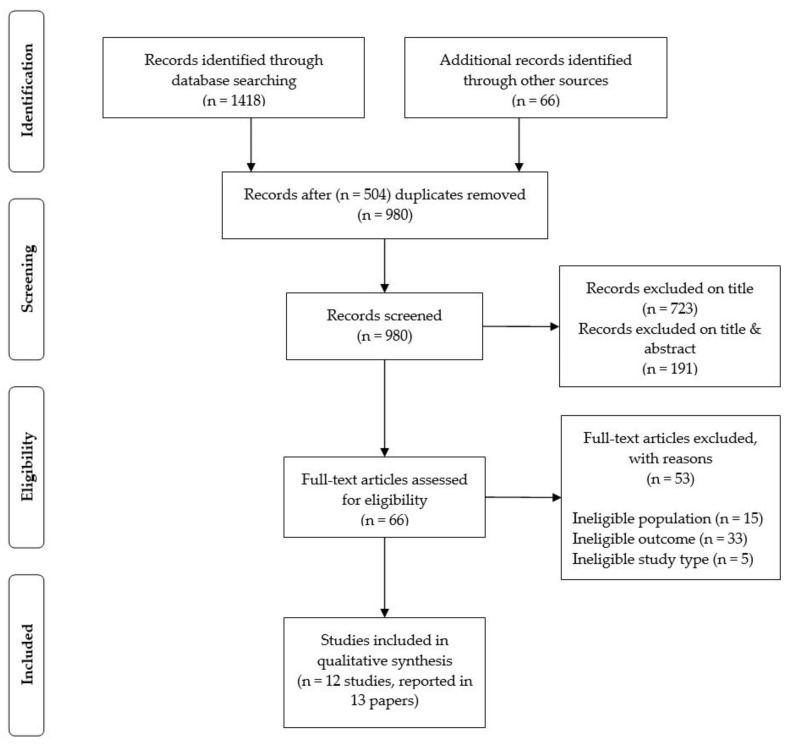
PRISMA flow diagram [32].

The systematic searches identified 980 records for screening by the reviewers plus 66 records from other sources (see Figure 1 for a depiction of the flow of information through the different phases of the review). A total of 12 studies reported in 13 papers were included in the review.

Most of the studies (*n* = 8) were conducted in Asian countries (five in China, one in India, one in Japan, and one in Iran), three were conducted in Canada (one study with Chinese-Canadian fathers (reported in two papers), and two with fathers of European, Asian, or Middle Eastern descent), and one in Turkey. Table 1 includes summaries of the seven articles that describe fathers’ views on facilitators and barriers associated with creating/maintaining a smoke-free home. Table 2 includes summaries of the six articles that assessed efforts to test smoke-free home interventions with a sample or sub-sample of fathers. Note: The number of studies in Table 1 and Table 2 are greater than 13 as one study [33] reported on barrier/facilitators and interventions. 

### 3.1. Facilitators and Barriers

Four of the seven studies identified that outlined fathers’ views on facilitators and barriers associated with creating/maintaining a smoke-free home were conducted in Asian countries (two in China, one in India, and one in Japan) [33,34,35,40], and three were conducted in Canada [36,37,38,39] (with one study reported in two papers [37,38]. Four studies used qualitative methods (focus groups, semi-structured face to face interviews, and telephone interviews) to explore the fathers’ views on creating/maintaining a smoke-free home [34,35,36,37,38]. Three of these studies had a wider study remit: to explore gender relations and masculinity in fathers who smoke [36]; fathers’ smoking behaviours [40], and fathers’ perspectives on stopping smoking [38]. One study used mixed methods (survey and focus groups) across different study phases [33], one was a quantitative study reporting findings from a cross-sectional survey [40], and one ethnographic study drew on interview transcripts, photographs that fathers had taken to document where their smoking took place both during and after their partner’s pregnancy, and field notes [39]. Four studies included mother and fathers [33,34,35,40] and three studies comprised exclusively of fathers [36,37,38,39].

#### 3.1.1. Beliefs and Knowledge

Beliefs and knowledge about SHS have the potential to enable or restrict the fathers’ attempts to create and maintain a smoke-free home. Inaccurate or incomplete knowledge about the health risks of SHS exposure can contribute to SHS exposure in children. In a qualitative study conducted in China [34], where approximately 60% of men and 7% women smoke, nine males (fathers/grandfathers) who were current smokers and one female (mother or grandmother, not specified) who was a non-smoker had misconceptions about SHS at home, believing that smoking in the living room or in the toilet does not lead to children being exposed to SHS. Participants (eight males who smoked, one female who smoked and six male non-smokers) thought that younger children were particularly at risk from SHS-related health issues because they were still developing, and six participants (four male smokers, one female smoker, and one female non-smoker) believed that once the child is older, their organs have developed and the risks are reduced, meaning that smoking in front of them is less harmful. The authors suggest that a lack of SHS-related knowledge on the part of smokers, mixed with Chinese social and cultural norms that are pro-smoking (see Section 3.1.2), has contributed to SHS exposure in children.

In quantitative survey work conducted in Kerala, India [33], where approximately 25% of men and 3% of women smoke, seventy percent of mothers surveyed from 140 households reported that their husband regularly smoked inside the house. Survey findings also indicated that fathers underestimated the risks associated with SHS exposure to children more often than mothers; 65% of mothers considered that SHS exposure could cause serious childhood illness compared to only 32% of fathers, and 28% of mothers believed it could cause minor illness or was harmless, compared to 42% of fathers.

In contrast, a qualitative study investigating the smoking-related experiences of immigrant Chinese fathers in Canada [37] highlighted the enabling role that knowledge can have in conjunction with becoming a father in a country where the message that SHS is harmful to pregnant women and young children had become commonplace. The 22 Chinese Canadian fathers in the study who smoked or had recently quit smoking reported that they had dramatically changed their smoking patterns because of concerns for their children’s health.

#### 3.1.2. Cultural and Perceived Social Norms

The Canadian study above [37] highlights how Chinese fathers could conform to dominant Canadian smoking norms and extend the ban on indoor smoking in public places into homes. It is well established that parents (mothers and fathers) with less formal education are more likely to smoke indoors, contributing to the social inequalities in home-smoking rates [4]. A Japanese cross-sectional survey suggested that parents who smoke with less formal education are more likely to perceive pro-smoking norms, which in turn may be associated with smoking in the home [40]. However, this study also found that household smoking status and worksite smoking status mediated the association between education and indoor smoking behaviours for fathers only. On this basis, the authors suggest that discouraging pro-smoking norms in the home and work social networks could help to reduce fathers’ smoking in the home.

#### 3.1.3. Gender Power Relations

Gender power relations within the household can enable or restrict the change to a smoke-free home where fathers smoke indoors. Nichter et al. [33] recognized the difficulty for individual women in Kerala, India to effect change in their household, developing a community-level rather than household-based smoke-free home intervention to change fathers’ smoking behaviour in the home as a result. Survey findings suggested that fathers were unwilling to change their smoking behaviour based on their wife’s or children’s requests not to smoke in the home. Findings from focus groups conducted with mothers suggested it was, in some circumstances, inappropriate but also potentially dangerous to challenge their husband’s home-smoking behaviour. Mothers also experienced difficulties in asking guests not to smoke in the home, which might be interpreted as disrespectful, given that it is culturally appropriate for men to smoke. Similar findings came from a recent qualitative Chinese study of families [34]. All 15 fathers who were current smokers reported that they smoked at home. Home-smoking restrictions were not discussed by families because of the social acceptability of smoking and authoritarian attitudes of the father or father-in-law. Three fathers effectively resisted their wives wishes for a smoke-free family home, with one saying, “*Every time I lit a cigarette at home my wife would complain, but I pretended that I did not to hear that she was talking. I knew she would stop her noise after sometime*.” [34] (p. 360) In contrast, findings from one qualitative study [35] with 13 fathers who were current smokers and 17 mothers who were non-smokers living in China with at least one child suggested that in most cases, mothers did have the authority to influence their husband’s home-smoking behaviour, although in a minority of cases, this was a sensitive issue. Many participants who had a smoke-free home policy had adopted it early on in their relationship. This often coincided with what the authors consider to be an ‘important opportunity’ before beginning a family, when men might be particularly invested in the health of their family in the home.

#### 3.1.4. Shifting Perceptions and Responsibilities Related to Fatherhood

In Canada, there is some evidence that changes in home-smoking patterns may be influenced by fatherhood. In one qualitative study [36] with 20 fathers, the majority described altering their home-smoking routines, with one reporting that smoking outside “*comes with the territory*” of fatherhood (p. 394). Gendered divisions of labour supported fathers’ smoking behaviours outside of the home, using chores including mowing the lawn and walking the dog as opportunities to keep their smoking outside. In some cases, being at home and involved in childcare reduced the fathers’ opportunities to smoke, a finding supported by the study of Chinese fathers who had moved to Canada [37]. The authors report that these fathers, conceding that their smoking could no longer be an autonomous decision, adopted a shift in masculine identity to that of ‘protector’, separating their smoking behaviours from family life to fulfil the contemporary role of the involved father. This was also the case in the Canadian study of new fathers [39], where fathers spoke of their preference to smoke at work rather than at home, as this gave them freedom to smoke without the surveillance from or risk to their child or partner. Some fathers linked the discussion of their outdoor smoking to notions of good fathering. Ten of the twenty participants had moved their smoking outdoors, although a minority reported that they broke house rules and secretly smoked indoors when the opportunity arose. Several participants were nostalgic for pre-fathering days when they had had the freedom to smoke inside the home.

### 3.2. Interventions

Of the six papers that assessed efforts to test smoke-free home interventions involving fathers, all but one were conducted in Asian countries (three in China, one in India, one in Iran) [33,41,42,43,44], with the remaining study conducted in Turkey [45]. Three were randomised controlled trials (RCT) assessing the feasibility of providing families with different counselling/educational interventions [41,44,45]. Two studies used a repeated cross-sectional design to assess the impacts of a ban on smoking in public places on home-smoking levels comparing pre-and post-ban survey data and home-smoking behaviour [42,43]. One study reported on assessing the feasibility of developing and delivering a community-based counselling/educational intervention across one proof of concept study and two pilot studies [33]. All six papers recruited both mothers and fathers to their sample. Three incorporated the objective assessment of SHS levels as an intervention component (two using infant urinary cotinine samples [41,45], and one using hair nicotine levels in mothers and children [43]).

#### 3.2.1. Counselling and Education

An RCT [41] in Iran demonstrated that counselling both fathers and mothers, alongside the receipt of an educational pamphlet and a sticker depicting a smoke-free home where the father chooses to smoke outside to protect his child, led to a significant reduction in exposure to SHS in the home (measured by cotinine and parental report) at the three month follow up. In Iranian families, cigarette smoking is not the cultural norm for women, reflected in the study sample whereby in 97% of households, only the father smoked. Fathers were viewed as not likely to be comfortable receiving instructions from their wives about refraining from smoking in the home, therefore in this cultural context, developing an intervention that directly encourages fathers to protect their children was considered important. Study findings suggested that despite these gendered cultural norms, a brief counselling program has the potential to change fathers’ home-smoking behaviour, at least in the short term. However, there was a lack of association between reported exposures and infant urinary cotinine levels, and the authors suggest that culture could play a role in the degree of accurate disclosure about smoking and SHS exposure.

#### 3.2.2. Education and Objective Assessment of Second-hand Smoke Levels in the Home

Presenting families with objective evidence on child SHS exposure using urinary cotinine levels, in conjunction with education materials, was shown to be an effective means of reducing SHS in the home in an RCT in Turkey [45]. In the intervention group, there were significant reductions in the number of cigarettes that fathers, but not mothers, smoked both daily and at home. Again, there was no correlation between child urinary cotinine levels and parentally reported SHS levels, suggesting that parents consciously or unconsciously did not acknowledge SHS exposure, or reported incomplete information. Of the children involved in the study, 81.4% showed signs of SHS exposure, and urinary cotinine levels were also high in the children of parents who claimed they never smoked in the home with children nearby. The authors suggest that such high levels may be explained by a lack of parental awareness, with parents not understanding that smoking at home, even when children are not present, can still lead to SHS exposure.

#### 3.2.3. Education and Mobile-Health Interventions

One RCT conducted in China (Changchun) [44] assessed whether an intervention that incorporated traditional and mobile-phone based education could assist families in creating a smoke-free home for infants. Non-smoking mothers of newborns and fathers received counselling at the same time, which facilitated spousal interaction and support, and encouraged the mothers to be the change agents. Mothers of the newborns in the intervention group reported reduced exposure to SHS at 12 months, suggesting that women have an important role to play in helping their spouses to change their smoking behaviour, as outlined in Section 3.1.3. However, the study relied on self-reporting to evaluate outcomes, which may be biased and inaccurate regarding SHS exposure levels in the home.

#### 3.2.4. Community-Based Interventions

In India, a community-based smoke-free homes initiative was developed, refined, and promoted across the state of Kerala, with the aim of reducing SHS exposure among mothers and children at home [33]. The initiative used a combination of healthcare worker household visits, and an educational video with positive messages to support fathers’ abstinence from smoking in the home as a sign of caring for women and children, and as a social value linked to the cultural value of male responsibility. The video also included testimonials from community members who had successfully created a smoke-free home as part of previous piloting of the initiative. At the baseline, across two pilot studies conducted in different communities, between 70–80% of fathers regularly smoked in the home, despite 80% of mothers having asked their husbands not to. Six months post intervention between 34% and 59% of fathers who smoked self-reported no longer smoking in the home. These observations were not validated by objective assessment of SHS levels.

#### 3.2.5. Impact of Smoke-Free Public Places Legislation

Two related studies investigated the impact of introducing smoke-free legislation in public places in Hong Kong on parental home-smoking behaviour [42,43]. In the first study, survey findings from the pre-and post-legislation groups found that significantly more fathers never smoked at home post-legislation (27% vs. 14%) and these differences remained significant after adjusting for the fathers’ educational level and age. The second study compared survey data with the assessment of SHS exposure levels, finding again that fathers smoking in the home significantly decreased post-legislation. Hair nicotine levels were lower in mothers and children post-legislation, and more mothers took action to protect their children from SHS including taking their children away from smoking and advising their husbands to quit. However, post-legislation, over 60% of the fathers reported that they still smoked at home when their children were not there, suggesting that more specific and effective smoke-free homes interventions are required in the future to assist fathers in changing their home-smoking behaviour.

## 4. Discussion

In this synthesis, we described studies that report on the role that beliefs/knowledge, cultural and perceived social norms, gender power relations and fatherhood play in fathers’ views on the creation and maintenance of a smoke-free home. We also highlighted the findings of interventions that have been developed to assist fathers to create a smoke-free home.

To date, whilst published systematic reviews and thematic syntheses of the smoke-free homes literature have incorporated studies including fathers as participants [10,46,47], the focus in most studies is on mothers’ experiences. Consequently, the barriers and facilitators that mothers face in creating/maintaining a smoke-free home are well documented, with gender imbalances visible through their lack of agency in effecting change in fathers’ smoking behaviours in the home [10]. A minority of interventions have been shown to reduce children’s exposure to SHS and improve children’s health, but the features that differentiate the effective interventions from those without clear evidence of effectiveness remain unclear [46]. The quality of evidence has been suggested to range from low to very low, and several suggestions have been made to address this in future interventions, for example by including more participants, describing interventions in more detail [46] and ensuring that objective outcomes are measured at baseline, at the end of the intervention, and at longer-term follow-up [47]. None of these suggestions include addressing the current gender imbalance inherent in smoke-free homes research, and yet increasing the involvement of fathers specifically could enhance our understanding of which interventions work for whom, and why. This would likely assist in improving our knowledge and understanding of the challenges that mothers and fathers face in reducing children’s exposure to SHS in the home, which could in turn lead to the development of interventions that more effectively engage all adult smoking household members.

The synthesis only identified 13 papers published over the last 10 years (written in the English language) that included findings on fathers’ roles in creating a smoke-free home, either through qualitative studies exploring barriers and facilitators, or through the development of interventions aiming to reduce children’s SHS exposure levels in the home. It is clear that cultural and gender norms play a significant role in shaping fathers’ beliefs and knowledge regarding the health risks of SHS exposure to children, and the importance associated with creating a smoke-free home. The extent to which the interventions outlined take account of the barriers and facilitators identified in this review varied. All but one of the intervention studies were conducted in Asian countries where smoking is not the cultural norm for women, and children are largely exposed to SHS through the father. Mothers may feel powerless to change their husband’s behaviour and fathers may not be willing to listen to requests to keep their smoking outside, due to the social acceptability of male smoking and/or authoritative attitudes. Most of the intervention studies conducted recognize this cultural context, and some acknowledge that it may lead to mothers over-reporting changes in fathers’ smoking behaviour in the home. Given these challenges, and in this cultural context, Nichter et al.’s [33] move away from an individual household intervention to the promotion of a community-wide intervention, with the end goal of changing smoking norms, shows promise. Their formative research also suggests that not smoking in the home could be effectively promoted as an important cultural value linked to male responsibility to protect the health of women and children, which fits with our finding that shifting perceptions and responsibilities related to fatherhood, and the role of the male as ‘protector’ [37,38,39] may facilitate fathers creating a smoke-free home. However, given the paucity of research conducted on fathers’ roles in creating a smoke-free home, further research is required to verify the utility of this approach.

A second approach that has shown initial promise is currently being trialled in Bangladesh to evaluate the effectiveness of a community-based intervention to reduce SHS exposure at home, primarily targeting men via mosques [48]. This cluster RCT is based on the findings of a pilot trial that concluded that a smoke-free home intervention was acceptable to Muslim communities, and feasible to deliver in mosques [49,50]. If found to be effective in changing smoking behaviour in the home, this approach could be generalizable to other communities with similar male smoking norms where faith-based settings (i.e., churches, mosques, synagogues) play an integral part in their lives [48].

Norms associated with male smoking differ significantly in Asian countries when compared to norms in the US, the UK, and other areas of Europe, where gender differences in smoking rates are less pronounced [51]. Little is known about the fathers’ roles and experiences of creating/maintaining a smoke-free home in these areas of the world, where gender equality may be a collective aspiration, for example, through the championing of initiatives such as shared parental leave. As most of the studies included in this scoping review were conducted in countries where gender inequality often exists, we acknowledge that approaches that are acceptable within these countries may not be generalizable to countries with a greater emphasis on gender equality. Different approaches may be required in Western European countries, for example, compared to Asian countries, to support fathers to effectively create and maintain a smoke-free home.

We were unable to source published research that considered the implications of different family/household compositions on creating a smoke-free home including fathers’ roles and experiences of negotiating smoke-free homes with an ex-partner to protect children who spend their time living between two parent’s homes. In addition, our searches found no studies that explored the role that fathers might play as agents of change in households where mothers smoke and fathers are non-smokers. A better understanding of fathers’ smoke-free home roles in different family compositions and smoking profiles, and the cultural context that their smoking behaviour operates within, could be considered as important to progress our understanding of the factors that lead to the creation and effective maintenance of a smoke-free family home. Despite the call for gender-sensitive and gender transformative approaches to tobacco control, these approaches have not been utilized in smoke-free homes research, which may have perpetuated the mothers’ responsibility for health in the home in this context, doing little to shift gender norms. Most of the studies identified in this review have focused on fathers’ individual roles and behaviours related to smoking in the home, without addressing some of the structural-level factors that shape masculinities (i.e., poverty, migration, racism, gender inequality) and give rise to smoking, and smoking in the home among fathers. The importance of structural interventions has been emphasized in relation to conducting gender-transformative work with men [52] because while men do have agency to make positive individual health changes, this agency sits in a wider social, economic, and cultural context that both constrains and enables individual choices [52,53]. Targeting this wider structural context could lead to more successful and fuller engagement with fathers in the future [52], which is key because effectively engaging fathers in the creation/maintenance of a smoke-free home has the potential to benefit the entire family, and improve gender equity as well as health.

Current smoke-free home intervention research involving fathers has been limited because of a lack of objective measurement of SHS exposure. One study conducted in China suggested that social desirability bias may affect some reporting of the prevalence of smoking in the home [34]. A study conducted in Iran has suggested that culture could play a role in the degree of accurate disclosure about smoking in the home [41]. A second Chinese study reported that even among participants (men and women with and without children) with a complete smoke-free home policy, 33% were exposed to SHS ‘in the past week’. Older relatives and visitors who smoke were reported as the key barrier to creating a smoke-free home, reflecting the Chinese value system whereby elder generations and visitors to the home are highly respected [54]. This finding builds on those of an earlier Chinese study conducted in six counties that found that 42% of non-smokers would offer cigarettes to guests visiting the home [55].

Introducing smoke-free public places legislation is associated with multiple child health benefits [56] including reduced exposure to SHS via increased home-smoking restrictions [57]. Two of the studies included in this scoping review suggest that the introduction of smoke-free legislation in Hong Kong may have assisted in reducing paternal smoking in the home [42,43], although 60% of the fathers involved in the Chan et al. [43] study reported that they still smoked at home when their children were not present. The challenge of capturing this fluidity of home-smoking rules in survey questions aiming to measure smoke-free home prevalence has been highlighted in previous research [10], and the importance of exploring fluidity as well as including objective measures of SHS exposure where practical, has been recommended in future intervention studies [10]. Consideration should also be given to the importance of developing theory-based smoke-free home interventions, as none of the studies described in this review paper had an explicit theoretical basis, and yet interventions based on theory are likely to be more successful than those non-theory based [58].

A key strength of this study lies in our comprehensive approach to conducting the scoping review. Triangulation was achieved by involving multiple authors in extraction, analysis, and interpretation of the results. Limiting our included data to English language papers may have biased the findings, and in scoping only published studies, it is possible that we missed other relevant research available as ‘grey’ literature. In addition, we screened potentially relevant papers by title and abstract only in the initial stages of the review process, which may have resulted in some papers with relevant findings documented in the full text of the paper being excluded, although our supplementary searches may have mitigated this somewhat. We also acknowledge that widening our inclusion criteria to include studies with expectant fathers may have revealed additional findings of relevance related to the specific context of a ‘teachable moment’. However, this was a small-scale review and our inclusion/exclusion criteria were agreed to on this basis. No quality appraisals were made of individual studies, as this was a rapid scoping review that adopted an inclusive approach.

## 5. Conclusions

Fathers have a central role to play in the creation and maintenance of a smoke-free family home. However, this scoping review highlights that very few published studies have (a) explored the barriers and facilitators associated with their role, or (b) developed and tested interventions designed to actively involve and appeal to fathers. The findings of this scoping review suggest that attitudes/knowledge, cultural and social norms, gender power relations. and shifting perceptions and responsibilities related to fatherhood could impact on the fathers’ views of their role in relation to creating/maintaining a smoke-free home, but additional research is required to support these suggestions, given the limitations of the scoping review already discussed. There are too few published intervention study findings that have focused on fathers’ roles in creating a smoke-free home to draw conclusions regarding effective approaches. However, in some Asian cultures where smoking is considered a social norm for men and not for women, a move away from individual household interventions to the promotion of a community-based intervention approach shows initial promise. However, research is also required in areas of the world where gender differences in smoking rates are less pronounced to begin to understand the fathers’ roles, successes, and challenges in creating and maintaining a smoke-free home, as effectively engaging fathers in the creation/maintenance of a smoke-free homes has the potential to not only benefit the entire family, but also improve gender equity and health.

## Figures and Tables

**Table 1:** Studies that describe fathers’ views on barriers and facilitators associated with creating/maintaining a smoke-free home.

Reference	Country	Purpose	Population	Study Design	Key Findings of Relevance to Fathers
Abdullah et al. 2012 [34]	China (Shanghai)	To explore attitudes to children’s exposure to second-hand smoke in the home in order to inform more effective smoke-free home interventions and policies.	A convenience sample of 31 caregivers (12 fathers, 10 mothers, 9 grandparents) with children aged 5 and under.	Qualitative study; 4 focus groups and 10 in-depth interviews. Thematic analysis.	Facilitators: Most participants said they were willing to protect their child from SHS exposure. Barriers: There was a lack of knowledge about the health risks of SHS exposure. Many families did not openly discuss smoking restrictions at home, and had no rules in place. Other barriers to adopting a smoke-free home included the social acceptability of smoking, hosting social gatherings at home, authoritative attitudes of the father or father-in-law, and difficulties with visitors who smoked.
Berg et al. 2011 [35]	China (Shanghai)	To examine the reasons, processes and challenges associated with establishing smoke-free homes policies.	13 fathers who were current smokers and 17 mothers who were non-smokers living with at least one child.	Qualitative study; 30 face to face semi-structured interviews. Thematic analysis.	Facilitators: Mothers were credited with initiating discussion regarding creating a smoke-free home more often and were reported to have decision making authority. Barriers: Common responses to their request to establish a smoke-free home among fathers were agreement, ignoring it, temporarily acquiescing, insisting on smoking in the home anyway, and devaluing the benefits of creating smoke-free homes. Challenges to enforcement included weather, social situations, the smoker being home alone, ineffective harm reduction behaviours such as smoking near windows, and addiction.
Kwon et al. 2014 [36]	Canada	To explore the role of masculinity in new and expectant fathers’ explanations of their continued smoking.	20 fathers (10 of European descent, and 10 of either Asian or Middle Eastern descent) from a previous study with a sample of 29 fathers.	Qualitative study; secondary analysis of interview data from a larger programme of research.	Facilitators: Most fathers reported reconciling with their partners to maintain a smoke-free home. In order to be responsible fathers and spousal partners, they accepted that their smoking routines needed to change. For some, new routines of parenting reduced their opportunities to smoke in the home. Domestic duties such as mowing the lawn and walking the dog provided them with opportunities to smoke outside. Fathers drew on masculine ideas such as protector and risk-taker, which influenced their smoking behaviour change efforts in the home.
Mao et al. 2015 [37], 2018 [38]	Canada (Ontario, Quebec, British Columbia)	To explore (1) the smoking-related experiences of immigrant Chinese fathers, and (2) the influence of denormalization in Canada on male Chinese immigrant smoking after migration.	22 fathers of Chinese origin who were currently smoking or had quit smoking in the past 5 years.	Qualitative study; semi-structured telephone interviews. Interpretive thematic analysis.	Facilitators: The message that exposure to SHS is harmful to pregnant women and young children was well understood. Fathers’ changes in smoking were constructed as voluntary behaviour modifications, rather than forced practices. The Chinese fathers were willing to conform to Canadian smoking norms and extended the ban on indoor smoking in the public sphere into homes. Becoming a father strengthened efforts to maintain a smoke-free home, even during the cold Canadian winter months. Involvement in childcare also increased the Chinese fathers’ determination to restrict their home smoking.
Nichter et al. 2015 [33] (see also Table 2)	India (Kerala)	To develop, refine and promote a community-based smoke-free homes intervention to reduce SHS exposure among women and children at home.	Survey: 140 husband wife pairs, where the husband was a smoker Focus Groups/Intervention development: 3 focus groups of 8 wives, whose husbands smoked.	Quantitative survey measuring attitudes re: SHS exposure.Qualitative; Focus groups discussing household gender relations and the ability of women to encourage a smoke-free home.	Barriers: Most women felt powerless to change their husband’s behaviour, as (typically in this region of India) husbands do not listen to advice from their wives about their personal habits. Men and women underestimated the risks of SHS exposure to child health, but men more so–65% of women thought it could cause serious illness, compared to only 32% of men. 28% of women believed it could cause minor illness or was harmless, compared to 42% of men.
Oliffe et al. 2010 [39]	Canada (Vancouver)	To investigate smoking and masculinities by detailing the highly gendered nature of the everyday places where fathers smoke.	20 new fathers who cohabited with their female partner and smoked during the pregnancy and postpartum period.	Ethnographic study–fathers took part in a semi-structured interview in the first month postpartum, were given a camera and encouraged to take pictures of the places that they smoked in during their partner’s pregnancy and afterwards. A second interview was then conducted to discuss photographs taken.	Facilitators: Most fathers understood the dangers of SHS exposure in the home. Fathers spoke of their preference to smoke at work rather than at home, as this gave them freedom to smoke without the surveillance from or risk to their child or partner. Some fathers linked the discussion of their outdoor smoking to notions of good fathering.
Saito et al. 2018 [40]	Japan	To test the potential mediating role of perceived smoking norms on the associations between education and indoor smoking among parents who smoke.	A convenience sample of 1645 parents (822 mothers, 823 fathers) from an online survey panel.	Quantitative; cross-sectional study.	Facilitators: Perceived smoking norms mediated the association between education and indoor smoking. Household smoking status and a worksite smoking ban also mediated this association via perceived norms, but only for fathers. Barriers: For both fathers and mothers who smoked, years of education was significantly negatively associated with indoor smoking behaviours.

**Table 2:** Studies that have assessed efforts to test smoke-free home interventions with a sample or sub-sample of men.

Reference	Country	Purpose	Population	Study Design	Key Findings of Relevance to Fathers
Baheiraei et al. 2011 [41]	Iran (Tehran)	To investigate whether counselling both mothers and fathers reduces their infants’ exposure to SHS.	N = 130 (convenience sample of families with children less than 1 year old, exposed to SHS. In 97% of households only the father smoked. Families were recruited whilst attending a health centre for routine infant health checks).	Randomised controlled trial. Mothers in the intervention group each received 3 counselling sessions, one of which was face to face (location not specified) and two of which were by telephone, and fathers in the intervention group received 3 counselling sessions by telephone. The control group received usual care.	In the intervention group, the number of smoke-free homes increased significantly from 15% at baseline to 33.3% at the 3-month follow-up. The differences between the two groups were statistically significant (*p* < 0.05). The intervention was effective in reducing infant urinary cotinine levels (*p* < 0.05).
Chan et al. 2011 [42]	China (Hong Kong)	To study whether smoking fathers would smoke inside their homes owing to smoke-free legislation in public places.	Pre-legislation group (2005) comprised of 186 families and the 2006 group of 114 families Post legislation group (2007a) comprised of 742 non-smoking mothers and 608 fathers and the 2007b group of 189 mothers, 174 fathers.	Prospective survey of two cohorts of families recruited before legislation and a cross-sectional survey of families after legislation.	Significantly more fathers in the 2007a group than the 2006 group never smoked at home (26.7% vs. 14.0%, *p* < 0.001), and never smoked around their children (59.7% vs. 30.7%, *p* < 0.001). The differences remained significant after adjusting for the father’s educational level and age. Regarding 60.6% of fathers who smoked at home and 45.3% of fathers who smoked around children in the 2007a group, they only smoked one to four cigarettes daily at home and around children, respectively.
Chan et al. 2014 [43]	China (Hong Kong)	To investigate the effect of maternal action to protect children from SHS and a 2007 public smoking ban, on children’s exposure to SHS in the home.	333 families participated in surveys prior to the smoking ban and 742 families participated in surveys post smoking ban.	Quantitative study, comparing survey data and direct measurement of SHS exposure levels from previous studies conducted prior to a public smoking ban, with that from survey data and SHS exposure levels collected for the present study post smoking ban.	Fathers’ smoking in the home decreased post-legislation. 29.3% of children post-legislation were exposed to SHS in the home, compared with 87.2% pre-legislation (*p* < 0.01). Hair nicotine level in mothers and children post-legislation was lower than pre-legislation. Over 90% of mothers pre-and post-legislation advised the fathers to reduce smoking, avoid smoking at home or avoid smoking near the children. This suggests that specific interventions for families should be expanded together with smoke-free legislation.
Nichter et al. 2015 [33] (see also Table 1)	India (Kerala)	To develop, refine and promote a community-based smoke-free homes intervention to reduce SHS exposure among women and children at home.	Proof of concept study: N = 140 Pilot study 1: N = 95 Pilot study 2: N = 157 (husband wife pairs, where husband was a smoker).	Community based intervention including educational meetings, smoke free homes video, healthcare worker household visits, community meetings and community declarations of support for smoke-free homes.	At baseline, across the pilot studies, between 70–80% of men regularly smoked in their home, despite 80% of women having asked their husband not to. Six months post intervention between 34% and 59% of men who smoked no longer smoked in their home. The authors note that this represents a modest, but significant change in community smoking norms. No statistical tests of significance were applied to the data.
Yu et al. 2017 [44]	China (Changchun)	To investigate if interventions that incorporate traditional and mobile phone based education help create smoke-free homes for infants and increase quitting among fathers.	N = 342 (families: non-smoking mothers and their newborns currently exposed to SHS in the home by fathers’ smoking).	Randomised controlled trial involving three groups: Intervention Group I-A received counselling on SHS harms to children, education on creating a smoke-free home, and posters to display in the home to encourage fathers and other visitors not to smoke. Intervention Group I-B received the same intervention as I-A, with additional text messages to the mother/father on harms of SHS to the mother and child. The father received additional text messages to quit smoking. Control Group: Received only standard care for their initial postnatal visits, which did not include any tobacco control or cessation counselling service.	Although no reduction of the self-reported exposure rate to SHS among surveyed mothers of newborns was found at 6 months, the rate at 12 months was significantly decreased in I-B compared to the control group. Participants in the I-B group were more likely to report “smoking never permitted inside home” compared to participants in control group at 12 months (1.17 vs. 4.71, *p* < 0.05). These findings suggest that the addition of an mHealth element to interventions with in-person counselling and provision of educational materials effectively aided in creating smoke-free homes among fathers of newborns.
Nacaroglu et al. 2017 [45]	Turkey (Izmir)	To determine whether informing families about their children’s urinary cotinine levels curtailed the exposure of children to SHS.	N = 193 children (Intervention group 97, control group 96). Families of the children recruited via a local hospital. There was no report of the family make-up and gender differences in the sample.	Randomised controlled trial. Urinary cotinine levels were measured in all children. Parents in the intervention group were given education about SHS harms and were advised about their child’s urinary cotinine levels by telephone. The control group were not informed about their child’s urinary cotinine levels until the end of the study.	In the intervention group, significant decreases in the number of cigarettes that fathers smoked both daily (16.8 to 14.5) and at home (7.69 to 3.96) were evident (*p* = 0.001 and *p* = 0.001, respectively). Although the number of cigarettes smoked daily by mothers both at home and outside decreased, the decreases were not significant.

## Supplementary Materials

The following are available online at https://www.mdpi.com/1660-4601/16/24/5164/s1, File S1: List of papers excluded at the full text screening stage.

## Appendix A

Sample search strategy

**Table A1:** Web of Science Core Collection (SCI-EXPANDED Science Citation Index Expanded; SSCI Social Sciences Citation Index; A&HCI Arts and Humanities Citation Index).

No.	Search String ^1^
#1	TS = (smok * OR tobacco OR cigarette * OR indoor * OR passive OR “ETS” OR “SHS” OR secondhand OR second-hand OR “second hand” OR antismoking OR “anti-smoking”)
#2	TS = (home$ OR “at-home” OR hous * OR accommodation$ OR “private” OR “privacy” OR resid * OR cohabit * OR co-habit *)
#3	TS = (father * OR step-father * OR stepfather * OR paternal OR husband * OR “male partner” OR “male partners” OR masculine *)
#4	#3 AND #2 AND #1
#5	#4 AND LANGUAGE: (English) AND Timespan = 2008–2019

^1^ TS searches the title, abstract, and keywords fields of a record; * represents any group of characters including no character; $ represents zero or one character.

Additional sources

When we compared our resulting list of included studies against those of other literature reviews on related topics (“The barriers and facilitators to smoking cessation experienced by women’s partners during pregnancy and the post-partum period: a systematic review of qualitative research” (Flemming et al. 2015 [29]) and “Gender, smoking and tobacco reduction and cessation: a scoping review” (Bottorff et al. 2014 [19])), we were interested to see that the studies that passed our inclusion criteria did not overlap much with the included studies. By examining the academic database indexing for the studies included by Flemming et al. (2015) and Bottorff et al. (2014), we found that records for these studies (usually titles, abstracts, and keywords) did not include terms for the ‘home’ concept from our search strategy, and a few did not include terms for the ‘fathers’ concept from our strategy. As the studies, broadly speaking, included fathers as a population and investigated smoking behaviours, we added the studies included in the two reviews to our pool of studies for further assessment. Many of the studies were authored by one or both of two Canadian academics, therefore we also ran a search for papers authored by Dr Joan Bottorff or Professor John Oliffe published since January 2008 that mentioned smoking/tobacco (Web of Science Citation Indices search string run on 19 August 2019: AU = (Bottorff OR Oliffe) AND TS = (smok * OR tobacco)). These additional sources added one study to our final results (Oliffe et al. 2010 [39]).
