# Supplementary material for: Fathers’ Views and Experiences of Creating a Smoke-Free Home: A Scoping Review
Source: Int J Environ Res Public Health. 2019 Dec 17;16(24):5164. doi: 10.3390/ijerph16245164 (PMC6950600; doi:10.3390/ijerph16245164)
Supplement: Supplementary file 1 [file ijerph-16-05164-s001.pdf]

Supplementary Materials

S.1 List of papers excluded at the full text screening stage

S1.1 Papers not meeting the Population criterion

1. Abdullah, A.S.; Driezen, P.; Sansone, G.; Nargis, N.; Hussain, G.A.K.M.; Quah, A.C.K.; et al. Correlates of exposure to secondhand smoke (SHS) at home among non-smoking adults in Bangladesh: findings from the ITC Bangladesh survey. *BMC Pulm Med* **2014**, *14*, 117. doi:10.1186/1471-2466-14-117.
2. Anuntaseree, W.; Mo-suwan, L.; Ma-a-lee, A.; Choprapawon, C. Prevalence and associated factors of passive smoking in Thai infants. *Prev Med* **2008**, *47* (4), 443–446. doi:10.1016/j.ypmed.2008.06.006
3. Aurrekoetxea, J.J.; Murcia, M.; Rebagliato, M.; Guxens, M.; Fernandez-Somoano, A.; López, M.J.; et al. Second-hand smoke exposure in 4-year-old children in Spain: Sources, associated factors and urinary cotinine. *Environ. Res.* **2016**, *145*, 116–125. doi:10.1016/j.envres.2015.11.028.
4. Bottorff, J.L.; Oliffe, J.L.; Kelly, M.T.; Greaves, L.; Johnson, J.L.; Ponic, P.; et al. Men's business, women's work: Gender influences and fathers' smoking. *Sociol Health Illn* **2010**, *32* (4), 583–596. doi:10.1111/j.1467-9566.2009.01234.x.
5. Bottorff, J.L.; Oliffe, J.L.; Sarbit, G.; Sharp, P.; Kelly, M.T. Smoke-free men: Competing and connecting to quit. *Am J Health Promot* **2018**, *32* (1), 135–142. doi:10.1177/0890117116671257.
6. Bottorff, J.L.; Oliffe, J.L.; Sarbit, G.; Sharp, P.; Caperchione, C.M.; Currie, L.M.; et al. Evaluation of QuitNow men: An online, men-centered smoking cessation intervention. *J. Med. Internet Res.* **2016**, *18* (4), e83. doi:10.2196/jmir.5076.
7. Bottorff, J.L.; Sarbit, G.; Oliffe, J.L.; Caperchione, C.M.; Wilson, D.; Huisken, A. Strategies for supporting smoking cessation among indigenous fathers: A qualitative participatory study. *Am J Mens Health* **2019**, *13* (1). doi:10.1177/1557988318806438.
8. Bottorff, J.L.; Sarbit, G.; Oliffe, J.L.; Kelly, M.T.; Lohan, M.; Stolp, S.; et al. "If I were Nick": Men's responses to an interactive video drama series to support smoking cessation. *J. Med. Internet Res.* **2015**, *17* (8), e190. doi:10.2196/jmir.4491.
9. Gould, G.S.; Munn, J.; Avuri, S.; Hoff, S.; Cadet-James, Y.; McEwen, A.; et al. "Nobody smokes in the house if there's a new baby in it": Aboriginal perspectives on tobacco smoking in pregnancy and in the household in regional NSW australia. *Women Birth* **2013**, *26* (4), 246–253. doi:10.1016/j.wombi.2013.08.006.
10. Herbert, R.J.; Gagnon, A.J.; O'Loughlin, J.L.; Rennick, J.E. Testing an empowerment intervention to help parents make homes smoke-free: a randomized controlled trial. *J Community Health* **2011**, *36* (4), 650–657. doi:10.1007/s10900-011-9356-8.
11. Kegler, M.C.; Haard◎rfer, R.; Berg, C.; Escoffery, C.; Bundy, L.; Williams, R.; et al. Challenges in enforcing home smoking rules in a low-income population: Implications for measurement and intervention design. *Nicotine Tob. Res*. **2016**, *18*, 976–981. doi:10.1093/ntr/ntv165.
12. Mantziou, V.; Vardavas, C.I.; Kletsiou, E.; Priftis, K.N. Predictors of childhood exposure to parental secondhand smoke in the house and family car. *Int J Environ Res Public Health* **2009**, *6* (2), 433–444. doi:10.3390/ijerph6020433.
13. Nichter, M.; Nichter, M.; Padmawati, R.S.; Ng, N. Developing a smoke free household initiative: an Indonesian case study. *Acta Obstet Gynecol Scand* **2010**, *89* (4), 578–581. doi:10.3109/00016340903578893.
14. Ossip, D.J.; Chang, Y.; Nabi-Burza, E.; Drehmer, J.; Finch, S.; Hipple, B.; et al. Strict smoke-free home policies among smoking parents in pediatric settings. *Acad Pediatr* **2013**, *13* (6), 517–523. doi:10.1016/j.acap.2013.06.003.
15. Sun, L.; Cheong, H.; Lee, E.; Kang, K.; Park, J. Affecting factors of secondhand smoke exposure in Korea: Focused on different exposure locations. *J. Korean Med. Sci.* **2016**, *31* (9), 1362–1372. doi:10.3346/jkms.2016.31.9.1362.

S1.2 Papers not meeting the Outcome criterion

1. Arghir, O.C.; Dantes, E.; Stoicescu, R.; Baicu, I.; Halichidis, S.; Ciobotaru, C.; et al. Parental environmental tobacco smoking and the prevalence of respiratory diseases in primary school children. *Pneumologia* **2013**, *62*(3), 178–181.

2. Berg, C.J.; Zheng, P.; Kegler, M.C. Family interactions regarding fathers' smoking and cessation in Shanghai, China. *J Smok Cessat* **2016**, *11* (4), 199–202. doi:10.1017/jsc.2014.25
3. Bottorff, J.L.; Kelly, M.T.; Oliffe, J.L.; Johnson, J.L.; Greaves, L.; Chan, A. Tobacco use patterns in traditional and shared parenting families: a gender perspective. *BMC Public Health* **2010**, *10*, 239. doi:10.1186/1471-2458-10-239.
4. Bottorff, J.L.; Oliffe, J.L.; Kelly, M.T.; Johnson, J.L.; Chan, A. Reconciling parenting and smoking in the context of child development. *Qual Health Res* **2013**, *23* (8), 1042–1053. doi:10.1177/1049732313494118.
5. Bottorff, J.L.; Oliffe, J.L.; Sarbit, G.; Kelly, M.T.; Cloherty, A. Men's responses to online smoking cessation resources for new fathers: The influence of masculinities. *JMIR Res Protoc* **2015**, *4* (2), e54. doi:10.2196/resprot.4079.
6. Bottorff, J.L.; Radsma, J.; Kelly, M.; Oliffe, J.L. Fathers' narratives of reducing and quitting smoking. *Sociol Health Illn* **2009**, *31* (2), 185–200. doi:10.1111/j.1467-9566.2008.01126.x.
7. Charalabopoulos, K.; Makris, G.; Charalabopoulos, A.; Golias, C.; Athanasiou, K. Public knowledge, beliefs and practices in Greece about cancer etiology and prevention. *East. Mediterr. Health J.* **2011**, *17* (5), 392–397.
8. de Carvalho Ribeiro, F.A.; de Moraes, M.K.; de Morais Caixeta, J.C.; da Silva, J.N.; Lima, A.S.; Parreira, S.L.; et al. Percepcao dos pais a respeito do tabagismo passivo na saude de seus filhos: um estudo etnografico. [Perception of parents about second hand smoke on the health of their children: an ethnographic study.] *Rev Paul Pediatr* **2015**, *33* (4), 394–399. doi:10.1016/j.rpped.2015.02.003.
9. Fernandez, M F.; Artacho-Cordon, F.; Freire, C.; Perez-Lobato, R.; Calvente, I.; Ramos, R.; et al. Trends in children's exposure to second-hand smoke in the INMA-Granada cohort: An evaluation of the Spanish anti-smoking law. *Environ. Res.* **2015**, *138*, 461–468. doi:10.1016/j.envres.2015.03.002.
10. Foulstone, A.R.; Kifle, T.; Kelly, A.B. The influence of partner smoking, relationship satisfaction and parental stress on tobacco use. *J Smok Cessat* **2018**, *14* (3) 155–160. doi:10.1017/jsc.2018.37.
11. Gage, J.D.; Everett, K.D.; Bullock, L. A review of research literature addressing male partners and smoking during pregnancy. *J Obstet Gynecol Neonatal Nurs* **2007**, *36* (6), 574–80. doi: 10.1111/j.1552-6909.2007.00188.x.
12. Greaves, L.; Oliffe, J.L.; Ponic, P.; Kelly, M.T.; Bottorff, J.L. Unclean fathers, responsible men: Smoking, stigma and fatherhood. *Health Sociol Rev* **2010**, *19* (4), 522–533. doi:10.5172/hesr.2010.19.4.522.
13. Huang, K.; Chen, H.; Liao, J.; Nong, G.; Yang, L.; Winickoff, J. P.; et al. Factors associated with complete home smoking ban among Chinese parents of young children. *Int J Environ Res Public Health* **2016**, *13* (2), 161. doi:10.3390/ijerph13020161.
14. Johnson, J.L.; Oliffe, J.L.; Kelly, M.T.; Bottorff, J.L.; LeBeau, K. The readings of smoking fathers: A reception analysis of tobacco cessation images. *Health Commun* **2009**, *24* (6), 532–547. doi:10.1080/10410230903104921.
15. Kayser, J.W.; Semenic, S. Smoking motives, quitting motives, and opinions about smoking cessation support among expectant or new fathers. *J Addict Nurs* **2013**, *24* (3), 149–157. doi:10.1097/JAN.0b013e3182a4caf1.
16. Kwon, J.Y.; Oliffe, J.L.; Bottorff, J.L.; Kelly, M.T. Masculinity and fatherhood: New fathers' perceptions of their female partners' efforts to assist them to reduce or quit smoking. *Am J Mens Health* **2015**, *9* (4), 332–339. doi:10.1177/1557988314545627.
17. Leung, D. Y.; Chan, S. S.; Mak, Y.; Leung, G.; Lam, T. Fathers' smoking behaviors at home and near their children after a smoke-free legislation in Hong Kong: A two-group comparison. *Circulation* **2010**, *122* (2), E274.
18. Loureiro, M. L.; Sanz-de-Galdeano, A.; Vuri, D. Smoking habits: Like father, like son, like mother, like daughter? *Oxf Bull Econ Sta*t **2010**, *72* (6), 717–743. doi:10.1111/j.1468-0084.2010.00603.x.
19. Mak, Y.W.; Loke, A.Y.; Abdullah, A.S.; Lam, T.H. Household smoking practices of parents with young children, and predictors of poor household smoking practices. *Public Health* **2008**, *122* (11), 1199–1209. doi:10.1016/j.puhe.2008.04.004.
20. Maloney, E.; Hutchinson, D.; Burns, L.; Mattick, R.P. Prevalence of and characteristics associated with persistent smoking among Australian mothers and fathers: Findings from the Longitudinal Study of Australian Children (LSAC). *J Fam Stud* **2010**, *16* (2), 165–175. doi:10.5172/jfs.16.2.165.

21. Morgan, E.H.; Graham, M.L.; Folta, S.C.; Seguin, R.AA qualitative study of factors related to cardiometabolic risk in rural men. *BMC Public Health* **2016**, *16*, 305. doi:10.1186/s12889-016-2977-1.
22. Oliffe, J.L.; Bottorff, J.L.; Sarbit, G. Supporting fathers' efforts to be smoke-free: Program principles. *Can J Nurs Res* **2012**, *44* (3), 64–82.
23. Oliffe, J.L.; Bottorff, J.L.; Kelly, M.; Halpin, M. Analyzing participant produced photographs from an ethnographic study of fatherhood and smoking. *Res Nurs Health* **2008**, *31* (5), 529–539. doi:10.1002/nur.20269.
24. Reid, M.C. The effects of family functioning and masculine ideologies on risky substance use. *Diss Abstr Int Section B* **2011**, *72* (2), 1155.
25. Robinson, J.; Ritchie, D.; Amos, A.; Cunningham-Burley, S.; Greaves, L.; Martin, C. 'Waiting until they got home': Gender, smoking and tobacco exposure in households in Scotland. *Soc Sci Med* **2010**, *71* (5), 884–890. doi:10.1016/j.socscimed.2010.04.031.
26. Saito, J.; Tabuchi, T.; Shibanuma, A.; Yasuoka, J.; Nakamura, M.; Jimba, M. 'Only fathers' smoking' contributes the most to socioeconomic inequalities: changes in socioeconomic inequalities in infants' exposure to second hand smoke over time in Japan. *PLoS ONE* **2015**, *10* (10), e0139512. doi: 10.1371/journal.pone.0139512.
27. Seong, M.; Moon, J.S.; Hwang, J.H.; Ryu, H.; Kang, S.J.; Kong, S.; et al. Preschool children and their mothers are more exposed to paternal smoking at home than school children and their mothers. *Clin. Chim. Acta* **2010**, *411* (1-2), 72–76. doi:10.1016/j.cca.2009.10.006.
28. Shiva, F.; Shamshiri, A.R.; Ghotbi, F.; Yavari, S.F. Exposure to secondhand smoke in infants: declining trends from 2001 to 2008? *Asia Pac J Public Health* **2011**, *23* (2), 157–162. doi:10.1177/1010539509339609.
29. Tautolo, E.; Iusitini, L.; Taylor, S.; Paterson, J. Will New Zealand be smokefree by 2025? Smoking prevalence amongst a cohort of Pacific adults. *N. Z. Med. J.* **2014**, *127* (1393), 99–106.
30. Tautolo, E.; Schluter, P J.; Taylor, S. Prevalence and concordance of smoking among mothers and fathers within the Pacific Islands Families Study. *Pac Health Dialog* **2011**, *17* (2), 136–146.
31. Tautolo, E.; Schluter, P.J.; Paterson, J.; McRobbie, H. Acculturation status has a modest effect on smoking prevalence among a cohort of Pacific fathers in New Zealand. *Aust N Z J Public Health* **2011**, *35* (6), 509–516. doi:10.1111/j.1753-6405.2011.00774.x.
32. Tsai, C.; Huang, J.; Hwang, B.; Lee, Y.L. Household environmental tobacco smoke and risks of asthma, wheeze and bronchitic symptoms among children in Taiwan. *Respir. Res*. **2010**, *11*, 11. doi:10.1186/1465-9921-11-11.
33. Zheng, Z.L.; Deng, H.Y.; Wu, C.P.; Lam, W.L.; Kuok, W.S.; Liang, W J.; et al. Secondhand smoke exposure of children at home and prevalence of parental smoking following implementation of the new tobacco control law in Macao. *Public Health* **2017**, *144*, 57–63. doi:10.1016/j.puhe.2016.11.018.

S1.3 Papers not meeting the Study Type criterion

1. Bielska, D.E.; Gomolka, E.; Kurpas, D.; Chlabicz, S. Narazenie dzieci w wieku przedszkolnym na dym tytoniowy--badanie porownawcze. [Exposure to environmental tobacco smoke in pre-school children--a comparative study.] *Prz. Lek*. **2015**, 72 (10), 505–508.
2. Bielska, D.; Trofimiuk, E.; Oldak, E.; Cylwik, B.; Chlabicz, S. Narazenie na dym tytoniowy a rodzaj ostrych infekcji ukladu oddechowego u dzieci. [Exposure to tobacco smoke and type of acute respiratory infections in children.] *Prz. Lek* **2010**, *67* (N10), 838–842.
3. Gomolka, E.; Bielska, D.; Nazarko, K.; Bielska, K. Narazenie dzieci w wieku przedszkolnym na dym tytoniowy i jego wplyw na stezenie kotyniny w moczu. [Kindergartners' indoor exposition to tobacco smoke and its influence on cotinine concentration in children's urine.] *Prz. Lek* **2013**, *70* (10), 818–821.
4. Vitali, M.; Protano, C. How relevant are fathers who smoke at home to the passive smoking exposure of their children? *Acta Paediatr*. **2017**, *106* (1), 74. doi:10.1111/apa.13659
5. White, C.; Oliffe, J.L.; Bottorff, J.L. Fatherhood, smoking, and secondhand smoke in North America: An historical analysis with a view to contemporary practice. *Am J Mens Health* **2012**, *6* (2), 146-155. doi:10.1177/1557988311425852.
